# Study on the Seismic Performance of Box-Plate Steel Structure with Openings Modular Unit

**DOI:** 10.3390/ma12244142

**Published:** 2019-12-10

**Authors:** Jinjie Men, Guanlei Fan, Tao Lan, Jiachen Wang, Liquan Xiong

**Affiliations:** 1 College of Civil Engineering, Xi’an University of Architecture & Technology, 13 Yanta Road, Xi’an 710055, China; menjinjie@yahoo.com (J.M.); fanguanlei2018@163.com (G.F.); wangjiachen2018@126.com (J.W.); xiongliquan2013@126.com (L.X.); 2 China Shipbuilding Industry Corporation International Engineering Co., Ltd., Beijing 100121, China

**Keywords:** box-plate steel structure with openings, seismic performance, failure mode, FEA, lateral force resistant capacity, calculation equation

## Abstract

The box-plate steel structure residence is a box structure with stiffened steel plates directly used as load-bearing walls and floors. In practical engineering, due to the functional requirements of the building, it is necessary to open door or window openings on the box-plate steel structure walls. To study the seismic performance of the box-plate steel structure with openings system, two three-story single-compartment box-plate steel structures with openings modular units were designed and fabricated according to the 1:3 reduced scale. Through the quasi-static loading test, numerical simulation, and theoretical analysis, the failure process, failure mode, lateral force resistant capacity, and hysteresis performance of the specimens were studied. The impact of the different opening areas and opening position on the seismic performance of the box-plate steel structure was emphatically analyzed. The results of the test indicated that the openings on the steel wall plate would reduce the initial stiffness and the lateral force resistant capacity of the specimen; the destruction of the box-plate steel structure with openings modular unit under the low cyclic loading effect started with the tear in the corner of the openings and ended with the tear in the corner steel wall plate. Then, the finite element analysis (FEA) models were developed to supplement the experimental study, and the comparisons were made between measured and simulated results on load versus displacement relationships and failure modes. On the basis of the stressing mechanism of the box-plate structure modular unit, the calculation equation of the lateral force resistant capacity of the box-plate structure with openings modular unit was put forward. Then, the proved finite element analysis (FEA) models were used for parameter analysis of different influence parameters to verify the proposed calculation equation. The results showed that the proposed calculation equation had high accuracy and could be used as a design basis for practical engineering.

## 1. Introduction

**Figure 1:**
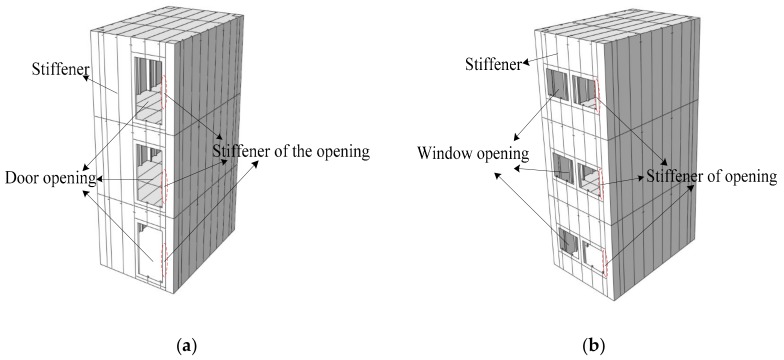
The schematic diagram of the box-plate steel structure modular unit with openings: (**a**) door openings; (**b**) window openings.

Steel plate shear wall (SPSW) is a new type of lateral force resistant structural member, developed in the 1970s, which is composed of steel plate wall, vertical edge member, and horizontal edge member [1,2,3]. The steel plate shear wall (SPSW) has the advantages of low cost, good seismic performance, and easy design [4,5,6,7]. At present, the basic principle of steel plate shear wall (SPSW) design [8,9,10,11,12] is based on the direct relationship between the capacity of the tension field after buckling of the thin plate and the total lateral load. The box-plate steel structure building [13] is a box structure with stiffened steel plates directly used as load-bearing walls and floors. The box-plate steel structures can be fabricated by modular prefabrication and assembled on site. This structure system has the advantages of a short construction period, integrated design and production, good seismic performance, flexible building space layout, large effective usable area, and good comprehensive economic benefits. Due to the need of building function, it is necessary to open door or window openings on the steel wall plate. Choi et al. [14] carried out the relevant experimental research and theoretical analysis on the steel plate wall with openings, and put forward the calculation formula of the lateral force resistant capacity of the steel plate shear wall with openings without stiffener; Vian et al. [15] conducted experiments and numerical analysis on the lateral force behavior of the shear wall with prefabricated non-stiffened steel plates with openings; Purba et al. [16] conducted numerical analysis on steel plate wall with openings, and gave suggestions on design of openings spacing and size, and gave the reduction of shear bearing capacity of steel plate wall caused by opening. In the previous literature review, research on the steel plate shear wall with openings was basically conducted at the component level, and few studies were conducted at the space stress-bearing unit level of the structure. In order to deeply understand the seismic performance of the box-plate steel structure with openings modular unit (Figure 1), the main works of this paper included: (1) two 1:3 reduced scale three-story single-compartment box-plate steel structures with openings modular units were tested under constant vertical compression and cyclically increasing lateral loading; (2) according to the results of experiment and the failure mode of the box-plate steel structure with openings modular unit, a simplified calculation method was proposed to evaluate the lateral force resistance capacity of the box-plate steel structure with openings modular unit; (3) the proved finite element analysis (FEA) models were used for parameter analysis of different influence parameters to verify the proposed calculation equation.

## 2. Experimental Program

### 2.1. Design of Specimens

In order to reduce the influence of boundary stiffness on the mechanical performance of space stress-bearing modular unit, a typical three-story single-compartment box-plate steel structure with openings modular unit was selected in the experimental design. Assuming that the shear force on the adjacent three stories was the same, the loading beam was set on the steel plate wall of the upper story, and the fixed end was set on the bottom of the lower story. In this way, the modular unit of the middle story was less affected by the boundary stiffness.

**Figure 2:**
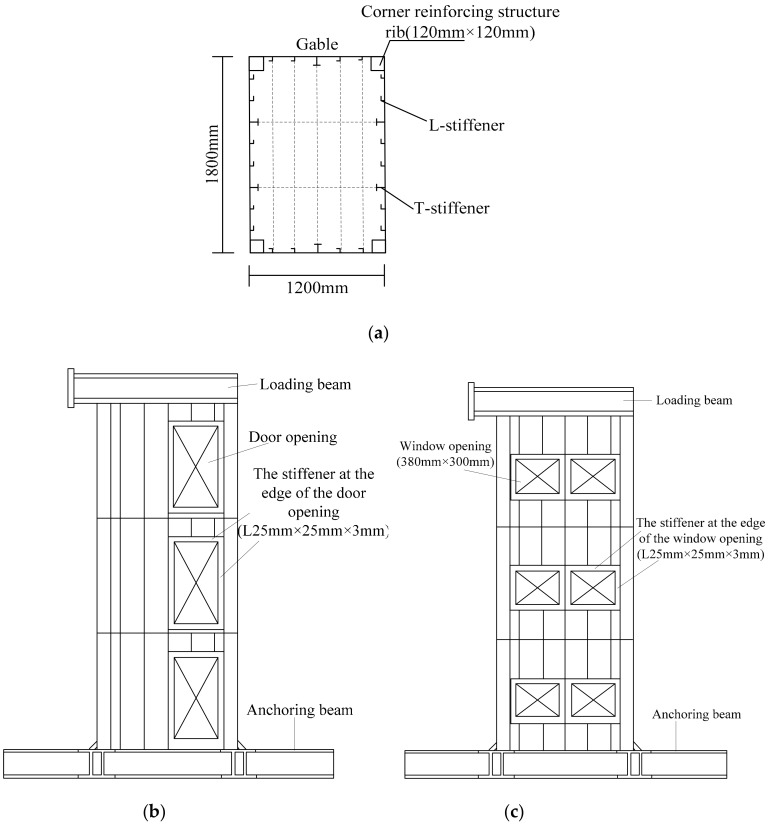
Construction of the two specimens: (**a**) cross section of specimen-1 and specimen-2; (**b**) elevation of specimen-1; (**c**) elevation of specimen-2.

The specimens were designed according to 1:3 of the prototype structure, and both the tow specimens were the same size. The size of the specimens was 1800 mm in length, 1200 mm in width, 1000 mm in story height, 3 mm in wall thickness, and 3 mm in plate thickness. A closed stiffener with a thickness of 5 mm and a size of 120 mm × 120 mm was set at the four corners of both specimens. The dimensions of stiffeners on walls and plates are shown in Table 1. Specimen-1 had one door opening with a size of 380 mm × 700 mm on each gable of each story, and specimen-2 had two window openings with a size of 380 mm × 300 mm on each gable of each story, as shown in Figure 2.

### 2.2. Material Test

**Figure 3:**
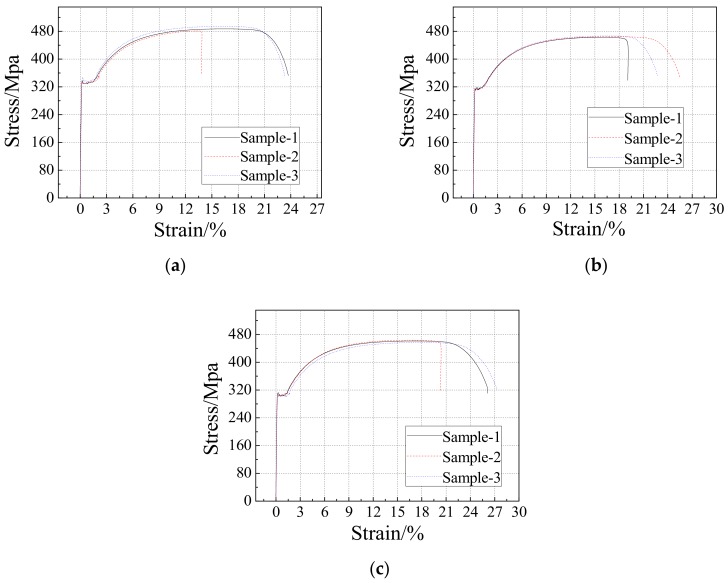
Material test of steel plate: (**a**) 3 mm thick steel plate; (**b**) 4 mm thick steel plate; (**c**) 5 mm thick steel plate.

The steel plate wall and stiffeners were made of Q235B steel. In the test, except for the loading beam and anchoring beam, each specimen involved three kinds of steel plates with a thickness of 3 mm, 4 mm, and 5 mm. Three samples were taken from each kind of steel plate with different thicknesses. The measured tensile stress-strain curve of steel plates of each thickness is shown in Figure 3. As could be seen from Figure 3, steel plates of three thicknesses had obvious elastic stages, yielding platforms, strengthening stages, and softening stages. The mechanical parameters of steel plates of each thickness were taken as the average value of the measured values of 3 samples, as shown in Table 2. The mechanical properties of the steel under uniaxial tension were determined by the material test. The test results are shown in Table 2.

### 2.3. Test Setup and Loading System

**Figure 4:**
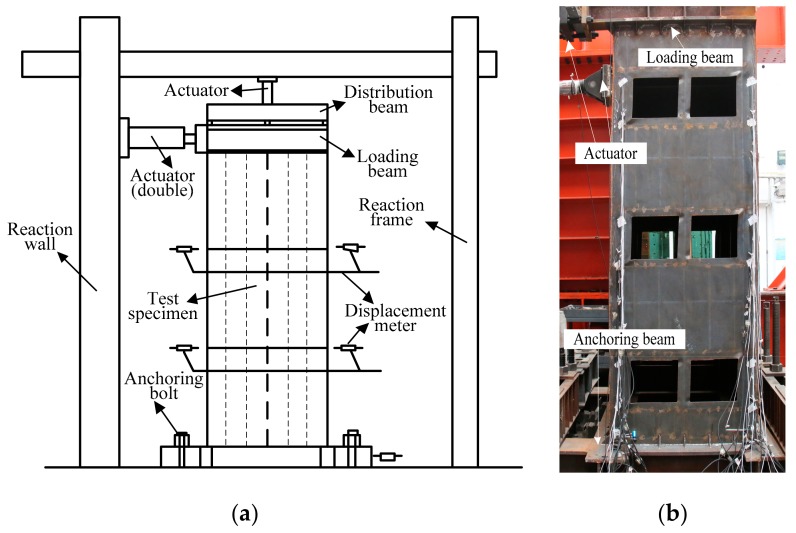
The layout of the test device and displacement measurement points: (**a**) sketch map; (**b**) actual loading diagram.

The anchoring beam of the specimens was fixed on the floor of the laboratory with anchoring bolts. A loading beam was welded to the top of the gable on both sides of the upper story of the specimens. One end of the loading beam was connected to the horizontal actuator during the test, and the vertical actuator distributed the vertical load to the loading beam on both sides through the distribution beam. A horizontal displacement meter was set at the two loading beams, respectively, to monitor the loading process; three horizontal displacement meters were set, respectively, at the floor of the second and third stories; a horizontal displacement meter was set at the anchoring beam at the bottom of the gable of the specimens to monitor the displacement of the support. The test device and the layout of displacement measurement points are shown in Figure 4.

**Figure 5:**
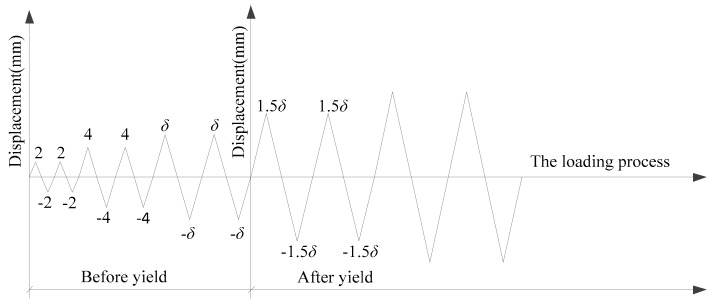
Loading system.

The vertical load was applied according to the axial compression ratio of 0.1, and then the horizontal load was applied with the vertical load unchanged. Two horizontal actuators were loaded synchronously. The displacement control method was adopted for reversed-cyclic loading. Each load level was cycled twice during the loading process. Before the yield of the specimens, the control displacement increment was 2 mm. After the specimens yielded, the 0.5 times of yield displacement was taken as an increment. The loading system is shown in Figure 5.

## 3. Failure Process and Failure Mode of the Specimens

### 3.1. Failure Process and Failure Mode of the Specimen-1

**Figure 6:**
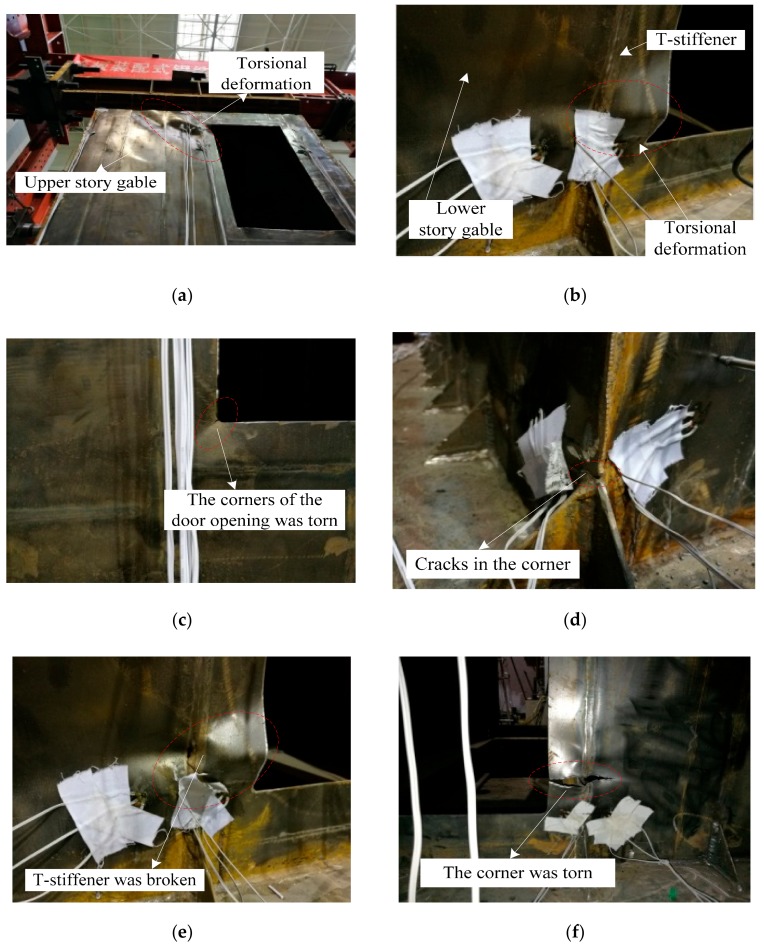
Failure process and failure mode of specimen-1: (**a**) T-stiffener had torsional deformation; (**b**) four corners of the door opening were buckled and deformed; (**c**) the corners of the door opening were torn; (**d**) cracks in the four corners of the specimen-1; (**e**) T-stiffener was completely broken; (**f**) the four corners of the specimen were torn.

At the beginning of loading, the specimen-1 was in the elastic stage. When the two horizontal actuators were loaded synchronously to the horizontal displacement of 8 mm, it could be known from the monitoring data of the strain gauges that the steel plate at the four corners of the door opening yielded. When the two horizontal actuators were loaded synchronously until the horizontal displacement was 42 mm, the four corners of the upper story door opening were buckled and deformed, and the T-stiffener at the middle position on the upper story gable had torsional deformation, as shown in Figure 6a. At this time, the maximum value of lateral force resistant capacity (pull direction) of specimen-1 was about 510 kN. When the two horizontal actuators were loaded synchronously until the horizontal displacement was 48 mm, the four corners of the lower story door opening were buckled and deformed, and the T-stiffener at the middle position on the lower story gable had torsional deformation, as shown in Figure 6b. At this time, the maximum value of lateral force resistant capacity (push direction) of specimen-1 was about 560 kN. When the horizontal displacement increased to 54 mm, the corners of the door opening of specimen-1 was torn (Figure 6c), and cracks appeared in the four corners of the specimen-1 (Figure 6d). At this time, the lateral force resistant capacity of the specimen-1 in both pushing and pulling directions began to enter the softening phase. When the two horizontal actuators were loaded synchronously until the horizontal displacement was 72 mm, the T-stiffener at the lower story of the gable was completely broken (Figure 6e), and the four corners of the specimen were torn (Figure 6f). At this point, the lateral force resistant capacity in both directions (push and pull) had decreased to below 85% of the maximum value, and the test was finished.

According to the phenomena and regularities of the specimen-1 during the whole process of loading, the failure mode of the box-plate steel structure with door openings modular unit specimen could be obtained as follows: the failure of the specimen-1 began with buckling deformation at the four corners of the door opening, and then the T-stiffener on the gable had buckling deformation, at which time the lateral force resistant capacity of the specimen-1 reached the maximum. Then, the four corners of the door openings were torn, and the corner of the whole specimen-1 was torn, and finally, the T-stiffener on the gable was broken.

### 3.2. Failure Process and Failure Mode of the Specimen-2

**Figure 7:**
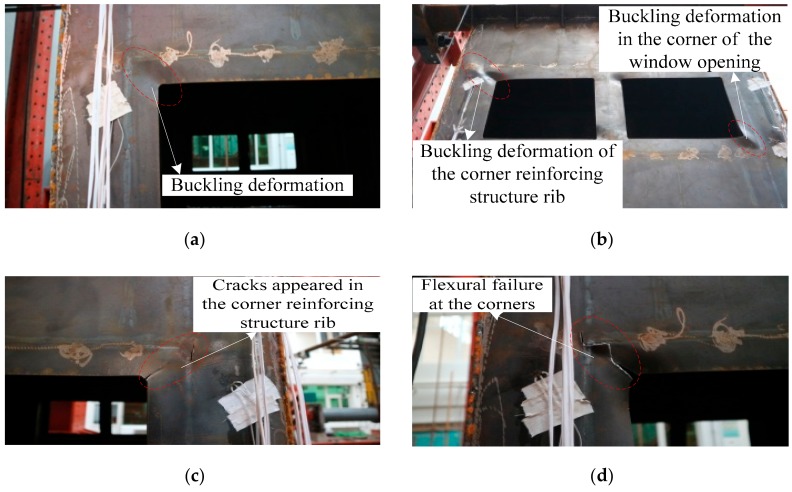
Failure process and failure mode of specimen-2: (**a**) the buckling deformation of the four corners of the window opening; (**b**) the corner reinforcing structure rib buckled; (**c**) cracks appeared in the corner reinforcing structure rib; (**d**) flexural failure of the specimen-2 at the four corners.

At the beginning of loading, the specimen-2 was in the elastic stage. When the two horizontal actuators were loaded synchronously to the horizontal displacement of 6 mm, it could be known from the monitoring data of the strain gauges that the steel plate at the four corners of the window opening yielded. When the horizontal displacement was 8 mm, the slope of the load versus displacement curve of the specimen-2 began to change, and the specimen-2 began to yield. When the horizontal displacement was 24 mm, the buckling deformation of the four corners of the window opening was obvious, as shown in Figure 7a. When the two horizontal actuators were loaded synchronously to the horizontal displacement of 48 mm, the cracks in the corner of the window opening of the specimen-2 developed further, and the buckling deformation of the corner reinforcing structure rib of the specimen-2 was obvious, as shown in Figure 7b. At this moment, the lateral force resistant capacity of the specimen-2 reached the maximum. When the horizontal displacement was 54 mm, cracks appeared in the corner reinforcing structure rib of the specimen-2, as shown in Figure 7c. At this time, the lateral force resistant capacity of the specimen-2 in both pushing and pulling directions began to enter the softening phase. When the two horizontal actuators were loaded synchronously to the horizontal displacement of 66 mm, the flexural failure of the specimen-2 at the four corners was observed, as shown in Figure 7d. At this point, the lateral force resistant capacity in both directions (push and pull) had decreased to below 85% of the maximum value, and the test was finished.

According to the phenomena and regularities of the specimen-2 during the whole process of loading, the failure mode of the box-plate steel structure with window openings modular unit specimen could be obtained as follows: the lateral force resistant capacity of the box-plate structure with window openings modular unit reached its maximum when cracks appeared in the corner of the window openings, and the buckling deformation appeared in the corner reinforcing structure rib. The damage of the box-plate structure with window openings modular unit began with the tearing at the corners of the window openings on upper and lower stories of the structure and the cracks at the corner reinforcing structure rib. When the box-plate structure with window opening modular unit was finally destroyed, the corner reinforcing structure ribs of the structure were torn, and the four corners of the structure were completely deformed.

By comparing the failure modes of the two specimens, the failure of the two specimens began with the buckling deformation of the steel plate at the corner of the openings. When the specimens failed, the four corners of the openings were torn, and the corner reinforcing structure ribs buckled. Because of the different area and position of the openings, the difference of lateral force resistant capacity of specimen-1 in two loading directions (push and pull) was larger than that of specimen-2. The plastic deformation of the steel plate wall was more uniform when specimen-2 was destroyed because the opening position of specimen-2 was symmetrical, and the specimen had the same lateral forces resistant capacity in both loading directions. The opening area of specimen-2 was larger, so the maximum bearing capacity of specimen-2 was less than that of specimen-1. However, due to the symmetrical position of the opening, specimen-2 had a stronger deformation ability.

## 4. Analysis of Experimental Results

### 4.1. Inter-Layer Shear Versus Inter-Layer Displacement Angle Response

**Figure 8:**
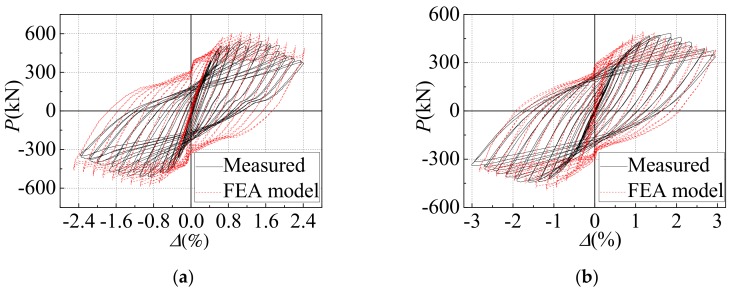
The inter-layer shear (*P*) versus inter-layer displacement angle (*Δ*) curves for the middle story of the two specimens: (**a**) Specimen-1; (**b**) Specimen-2.

**Figure 9:**
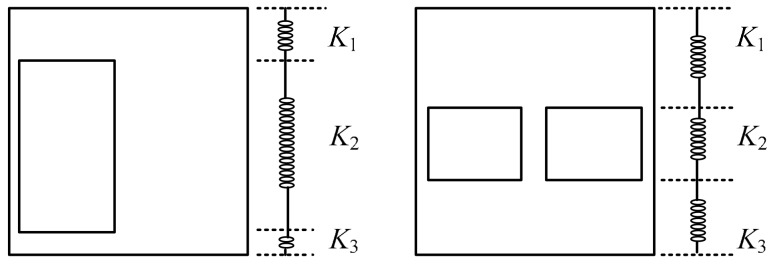
Stiffness composition of steel plate.

In order to reduce the influence of boundary stiffness on the mechanical performance of space stress-bearing modular unit, Figure 8 shows the inter-layer shear (*P*) versus inter-layer displacement angle (*Δ*) curves for the middle story of the two specimens. Before the specimen-1 and the specimen-2 yielded and flexed on both sides of the gable, the inter-layer shear (*P*) versus inter-layer displacement angle (*Δ*) curves were basically linear, and the area surrounded by the hysteresis loop was small, and the specimens were in an elastic working state. As the inter-layer displacement angle (*Δ*) increased, partial yielding and buckling occurred in the steel plate around the gable openings on both sides. The hysteresis curve was gradually inclined to the horizontal axis, and the area surrounded by the hysteresis loop increased, the stiffness degraded, and the specimens entered the elastic-plastic working stage. Due to the tearing of the corner of the specimens and the torsional deformation of the gable of specimens, the lateral force resistant capacity was declining until the specimens were destroyed. The push direction of the lateral force resistant capacity of specimen-1 was higher than that of the pull direction. The reason was that the position of the door openings was asymmetrical, the gable wall plate was close to the side of the horizontal actuator, and the door openings were away from the horizontal actuator side. When the horizontal actuator was pushed, the corner reinforcing structure ribs were pulled near the gable wall plate. The wall plate was more embedded, yet when the horizontal actuator was pulled, the corner reinforcing structure ribs near the gable wall plate were pressed, and the wall plate was less embedded. The specimen-2 had a uniform opening position, so the hysteresis curve was more symmetrical than the specimen-1. Within the height range of opening on each story gable wall plate, the sheared section area of the specimen-2 was smaller than that of the specimen-1, so the maximum value of lateral force resistant capacity of the specimen-2 was smaller than that of the specimen-1. The initial stiffness of specimen-2 was less than that of specimen-1. This was because the elastic lateral stiffness of steel plate wall with openings could be regarded as the lateral stiffness of steel plate wall at the upper part of the opening (*K*_1_), the lateral stiffness of steel plate wall at the height of the opening (*K*_2_), and the lateral stiffness of steel plate wall at the lower part of the opening (*K*_3_) in series, as shown in Figure 9. Compared with *K*_2_ of specimen-1, *K*_2_ of specimen-2 weakened more seriously, so the total initial stiffness of specimen-1 was greater than that of specimen-2.

### 4.2. P-Δ Envelope Curve

**Figure 10:**
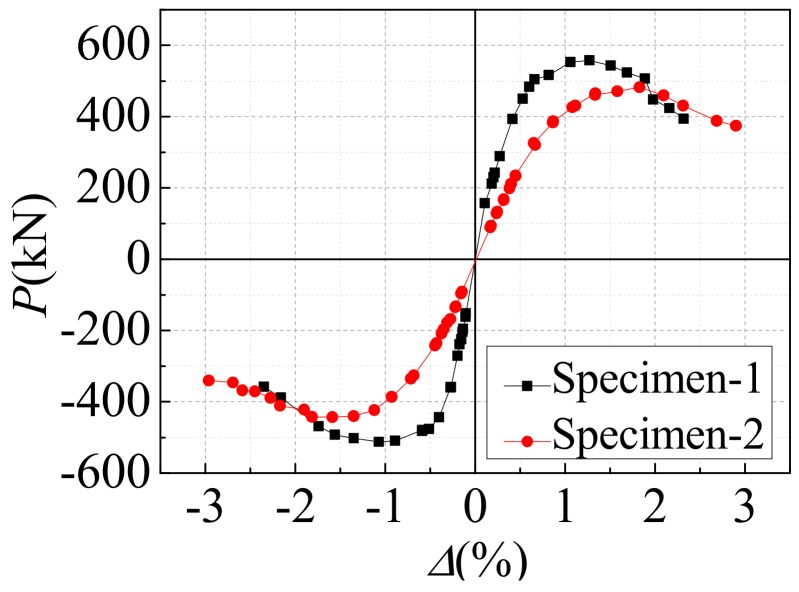
*P-Δ* envelope curves.

Figure 10 exhibits the *P-Δ* envelop curves of the specimens. These *P-Δ* envelop curves in the elastic phase were basically linear. Obviously, the initial stiffness of specimen-2 was significantly less than that of specimen-1. This was because, within the height range of the window openings on the gable of specimen-2, the area of the openings accounted for the largest proportion of the area of the steel wall plate. The difference of peak bearing capacity between the two directions (push and pull) of specimen-1 was larger than that of specimen-2, which was caused by the asymmetry of the opening position of specimen-1. In general, the *P-Δ* envelop curves of both specimens experienced the stages of elastic rise, elastic-plastic strengthening, and decline, and both specimens had good ductility and the lateral force resistant capacity. The maximum inter-layer displacement angle of both specimens exceeded 2%, which met the seismic requirements. Table 3 shows the primary performance indicators of the two specimens. From Table 3, it could be obtained that the ultimate bearing capacity of specimen-1 under push loading was 558.09 kN, and that under pull loading was 512.5 kN. In addition, the ultimate bearing capacity of specimen-2 under push loading was 482.59 kN, and that under pull loading was 443.23 kN.

### 4.3. Energy Dissipation

**Figure 11:**
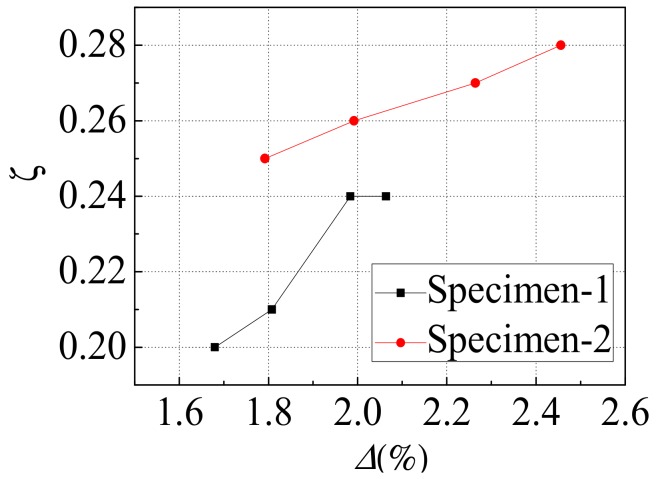
Energy dissipation.

Figure 11 exhibits the equivalent viscous damping ratio (*ζ*) versus inter-layer displacement angle (*Δ*) curves for the middle story of the two specimens. The equivalent viscous damping ratios of the two specimens both showed an increasing tendency as the inter-layer displacement angle increased, and the equivalent viscous damping ratio of the specimen-2 was greater than that of specimen-1. This was because the window openings in the gable of specimen-2 weakened the strength and stiffness of the steel plate more than that of specimen-1, and made the anchoring effect of the corner reinforcing structure ribs of specimen-2 on the steel plate better than that of specimen-1. Compared with specimen-1, although the initial stiffness and the lateral force resistant capacity of specimen-2 were lower than that of specimen-1, specimen-2 had better energy dissipation capacity and deformation capacity.

### 4.4. Stiffness Degradation

**Figure 12:**
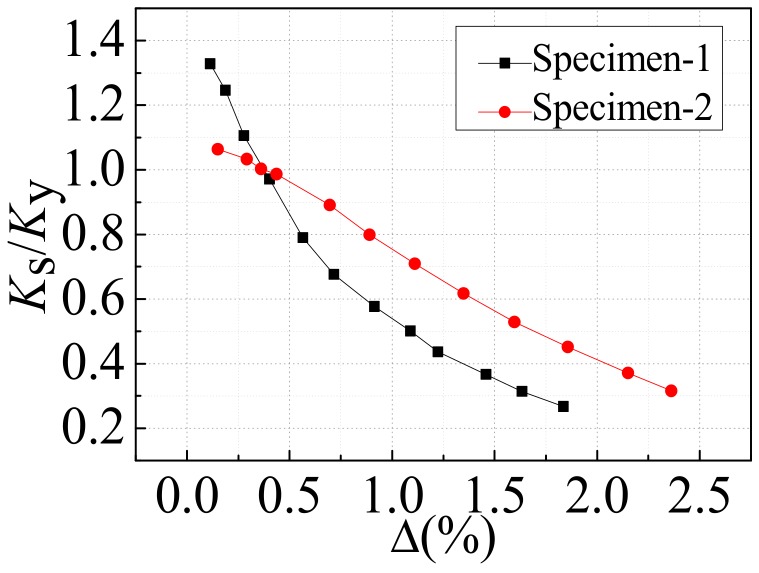
Stiffness degradation.

The stiffness of the specimen can be expressed by secant stiffness (*K_S_*), which is expressed as follows:

(1) $$K_{s}=\frac{\left|+P_{1}\right|+\left|-P_{1}\right|}{\left|+\Delta_{1}\right|+\left|-\Delta_{1}\right|}$$

where *P*_1_ and *Δ*_1_, respectively, represent the peak load and corresponding displacement of the first cyclic load in each loading stage. The secant stiffness of each loading stage (*K_S_*) was divided by the secant stiffness of the specimen at yield (*K_y_*), and the secant stiffness was dimensionless. The stiffness degradation curves of the two specimens are shown in Figure 12. As could be seen from Figure 12, the initial stiffness of specimen-1 was larger than that of specimen-2, mainly because the area of the opening on the gable steel plate of specimen-1 was smaller. The stiffness degradation rate of specimen-1 was relatively fast before yield and gradually slowed down after yield, mainly because the plastic deformation of specimen-1 increased after yield, which could dissipate more input energy. Compared with specimen-1, the stiffness degradation of specimen-2 was more uniform and stable. After yielding, specimen-2 still had a secant stiffness close to yielding stiffness, indicating that specimen-2 had a stable bearing capacity.

### 4.5. Strength Degradation

**Figure 13:**
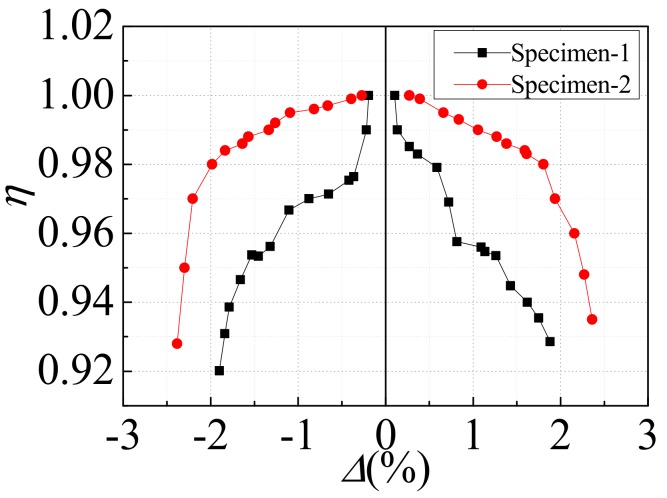
Strength degradation.

The strength degradation coefficient (*η*) is adopted to represent the strength degradation of the specimen, where the strength degradation coefficient (*η*) means the ratio of the peak point load of the last cycle to the peak point load of the first cycle under the same displacement amplitude. Figure 13 exhibits the strength degradation coefficient (*η*) versus the inter-layer displacement angle (*Δ*) curve of the two specimens. It could be seen from Figure 13 that during the whole loading process, the strength degradation of specimen-1 was more serious than that of specimen-2, which also verified that specimen-2 had better ductility. This was because the opening position of specimen-1 was asymmetric, and the bearing capacity of the two directions was different during cyclic loading. When the inter-layer displacement angle of specimen-1 exceeded 1%, the strength degradation was intensified, which was mainly due to the buckling of the T-stiffener rib, and the plastic deformation of specimen-1 increased sharply. The strength degradation of specimen-2 was relatively stable. When the inter-layer displacement angle was less than 2%, the strength degradation coefficient was above 0.98. When the inter-layer displacement angle of specimen-2 exceeded 2%, the strength degradation coefficient decreased rapidly, which was mainly due to the buckling deformation of the corner reinforcing structure rib, the bearing capacity of specimen-2 decreased, and the steel plate at the corner of the opening was torn. In general, the strength degradation coefficients of the two specimens were above 0.92, and the strength degradation was relatively slow, with a good safety reserve.

## 5. Finite Element Analysis and Prediction of the Ultimate Shear Capacity (*Vu*)

### 5.1. Finite Element Model

**Figure 14:**
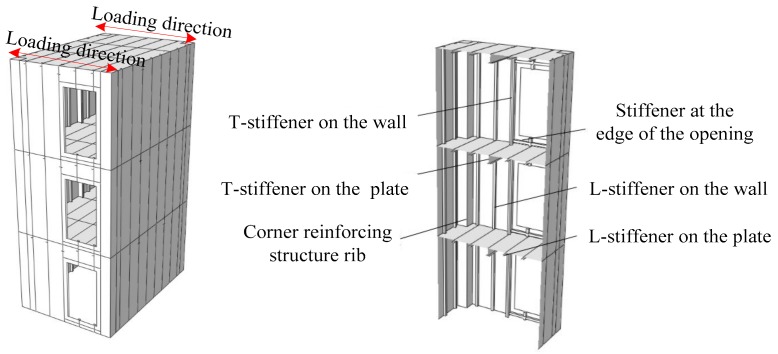
Finite element model.

In order to verify the effectiveness of the experiment, finite element software ABAQUS (version 6.14) was used to carry out finite element analyses on the seismic performance of the above two specimens. All the components in the model were modeled with shell element S4R, as shown in Figure 14. The constitutive structure of steel was made of ideal elastoplasticity. According to the results of the materiality test, the elastic modulus of steel was 2.21 × 105 MPa, and the yield strength was 331 Mpa. The material properties are shown in Table 1. In order to ensure the convergence of the calculated results, the quadrilateral structure division method was used to divide the mesh of the model after all the cells were neatly segmented. The vertical load was converted to the uniformly distributed load applied to the steel plates within the width of the loading beams on both sides. The bottom of the model was directly set as embedding, so as to achieve the embedding effect of the specimen welded on the anchoring beam in the experiment. The maximum value of the initial geometric imperfection was *λ*/500, where *λ* was the slenderness ratio of the single-story gable steel plate. The material properties and loading law were consistent with the previous tests. The ABAQUS/Explicit analysis module was adopted for the solution.

### 5.2. Finite Element Verification

#### 5.2.1. Comparison of Hysteresis Curves

Comparisons between the measured and simulated inter-layer shear (*P*) versus inter-layer displacement angle (*Δ*) relations are shown in Figure 8. The hysteresis curves obtained by simulation were fuller than that measured by a test, and the loading stiffness and unloading stiffness were both higher. This was because the materials in the FEA model were uniform. It was impossible to accurately simulate the tearing failure of steel plate. However, in general, the pinching phenomenon was well captured, and the FEA models reached a reasonable agreement with the measured results for the two specimens. So, this finite element analysis method could be used to conduct a more in-depth analysis of the two specimens.

#### 5.2.2. Comparative Analysis of Failure Process and Failure Mode

**Figure 15:**
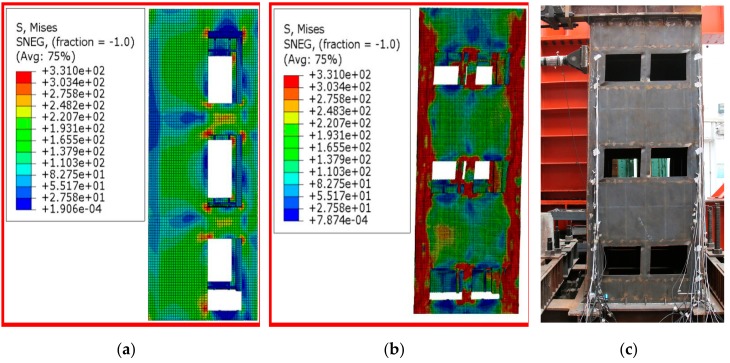
Comparative analysis of failure process and failure mode of specimen-1: (**a**) Mises stress cloud image of specimen-1 at yield; (**b**) Mises stress cloud of specimen-1 at final failure; (**c**) Test photo of specimen-1 when it was damaged.

**Figure 16:**
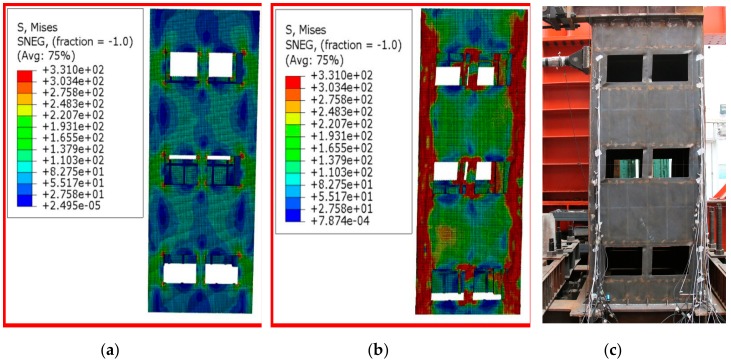
Comparative analysis of failure process and failure mode of specimen-2: (**a**) Mises stress cloud image of specimen-2 at yield; (**b**) Mises stress cloud of specimen-2 at final failure; (**c**) Test photo of specimen-2 when it was damaged.

Comparisons of FEA model simulation and test in the failure of the two walls are shown in Figure 15 and Figure 16. As can be seen from Figure 15, the finite element simulation of specimen-1 was basically consistent with the failure process and failure mode obtained by the test. When the horizontal displacement was 8 mm, the Mises stress cloud image of specimen-1 was observed, as shown in Figure 15a, which was in good agreement with the results of the test that the steel plate with door opening first yielded during the test. With the increase of horizontal displacement, the shear force on the gable wallboard of specimen-1 increased gradually. Figure 15b shows the Mises stress cloud image of specimen-1 at its final failure. The comparison between Figure 15b,c showed that the finite element simulation and experimental results were in good agreement.

Figure 16a shows the Mises stress cloud image of specimen-2 with the loaded horizontal displacement of 6 mm. It could be seen from the figure that the steel plate with the window opening first yielded, which was consistent with the test process. Figure 16b shows the Mises stress cloud image of specimen-2 at the final failure. According to the stress distribution in Figure 16b, the failure of the specimen-2 was concentrated in the window opening and the four corners of specimen-2, which was in good agreement with the failure mode of specimen-2 at the end of the test, as shown in Figure 16c.

### 5.3. Prediction of the Ultimate Lateral Force Resistant Capacity (Vu)

**Figure 17:**
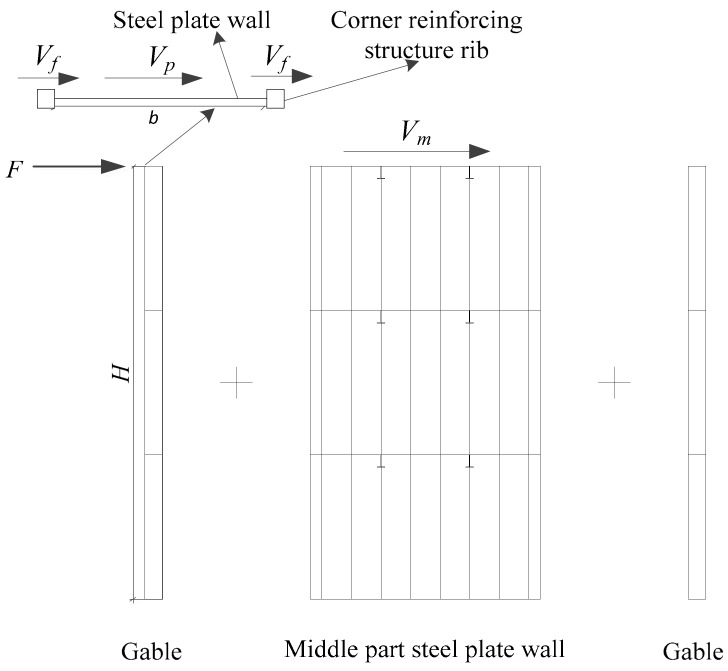
Calculation of model.

Mao et al. [13] studied the influence parameter and the calculation method of a three-story single box-plate steel structure space stress-bearing modular unit with corner reinforcing structure rib. The key factors affecting the mechanical performance of box-plate steel structure modular units were axial compression ratio (*n*), the flexibility coefficient of corner reinforcing structure rib (*β*), and the height-thickness ratio of steel plate (*λ*). The larger *λ*, the larger *β,* and the smaller *n* could all improve the carrying capacity of the modular unit, where the influence of *n* was relatively small. The ultimate lateral force resistant capacity of the three-story single box-plate steel structure space stress-bearing modular unit with corner reinforcing structure rib (*V_u_*) could be approximately considered to be composed of three parts: (1) the ultimate lateral force resistant capacity of the corner reinforcing structure rib (*V_f_*); (2) lateral force resistant capacity of gable steel plate in full section shear yield (*V_p_*); (3) the shear force born by the middle part steel plate wall (*V_m_*), as shown in Figure 17. The equation could be written as follows:

(2) $$V_{u}=2(V_{f}+V_{p})+V_{m}$$

Ignoring the flexural stiffness of the story in the gable steel plate, *V_f_* was the ultimate lateral force resistant capacity when the corner reinforcing structure rib of the model reached the ultimate bending moment. The *V_f_* could be calculated using the following equation:

(3) $$V_{f}=(4M_{c}-2N\delta )/H$$

where *M_c_* was the ultimate bending moment of the corner reinforcing structure rib under the action of bending and compression; *N* was the vertical pressure of the corner reinforcing structure rib at the top of the three-story single box-plate steel structure space stress-bearing modular unit; *δ* was the lateral displacement of the model under the ultimate load; *H* was the distance between the upper horizontal action of the three-story single box-plate steel structure space stress-bearing modular unit and the bottom section of the model.

The results of the experiment and finite element simulation showed that the steel plate of the gable yielded first and then buckled; therefore, the lateral force resistant capacity of the gable steel plate was the yield of the whole section of the gable steel plate. So, the lateral force resistant capacity of gable steel plate in full section shear yield (*V_p_*) could be calculated as follows:

(4) $$V_{p}=\tau_{y}bt_{w}=(f_{y}/\sqrt{3})bt_{w}$$

where *b* was the width of the gable steel plate; *t_w_* was the thickness of the steel plate; *f_y_* was the yield strength of materials.

The shear force born by the middle part steel plate wall (*V_m_*) could be calculated as follows:

(5) $$V_{m}=0.3f_{y}A_{m}b_{m}$$

where *A_m_* was the area of single middle part steel plate wall; *b_m_* was the distance between the two single middle part steel plate wall.

For the three-story single box-plate steel structure with openings space stress-bearing modular unit, the calculation model of ultimate lateral force resistant capacity (*V_u_*_,0_) was consistent with that of three-story single box-plate steel structure space stress-bearing modular unit. Because of the opening in the gable, the bearing capacity of the gable steel plate under shear yield was different from that of the structure without opening. It was assumed that *V_p_*_,0_ was the lateral force resistant capacity of the full-section of the gable steel plate with opening subjected to shear yield. Nie et al. [17], through finite element calculation and parameter analysis, proposed that *V_p_*_,0_ could be calculated by the following equation:

(6) $$\left\{\begin{array}{l} V_{p,0}=w_{f}V_{p}\\ w_{f}={\left(1-\chi^{\xi }\right)}^{\alpha }\\ \chi =\frac{A_{0}}{A} \end{array}\right.$$

(7) $$\alpha =-0.553{\left(\frac{h}{b}\right)}^{2}+1.93(\frac{h}{b})+0.989$$

(8) $$\xi =\left\{\begin{array}{l} 0.05{\left(\frac{h_{0}}{b_{0}}\right)}^{2}-0.094(\frac{h_{0}}{b_{0}})+1.089,(\frac{h_{0}}{b_{0}}\geq 1)\\ 1.04-0.04(\frac{h_{0}}{b_{0}}),(\frac{h_{0}}{b_{0}}<1) \end{array}\right.$$

where *A*_0_ was the opening area of the gable steel plate; *A* was the area of the gable steel plate; *χ* was the opening rate of the gable steel plate; *h* was the depth of the gable steel plate; *h*_0_ was the depth of the opening; *b* was the width of the gable steel plate; *b*_0_ was the width of the opening.

So, the ultimate lateral force resistant capacity (*V_u_*_,0_) of the three-story single box-plate steel structure with openings space stress-bearing modular unit could be calculated as follows:

(9) $$V_{u,0}=2(V_{f}+V_{p,0})+V_{m}$$

### 5.4. Verification and Analysis of the Ultimate Lateral Force Resistant Capacity Calculation Equation

**Figure 18:**
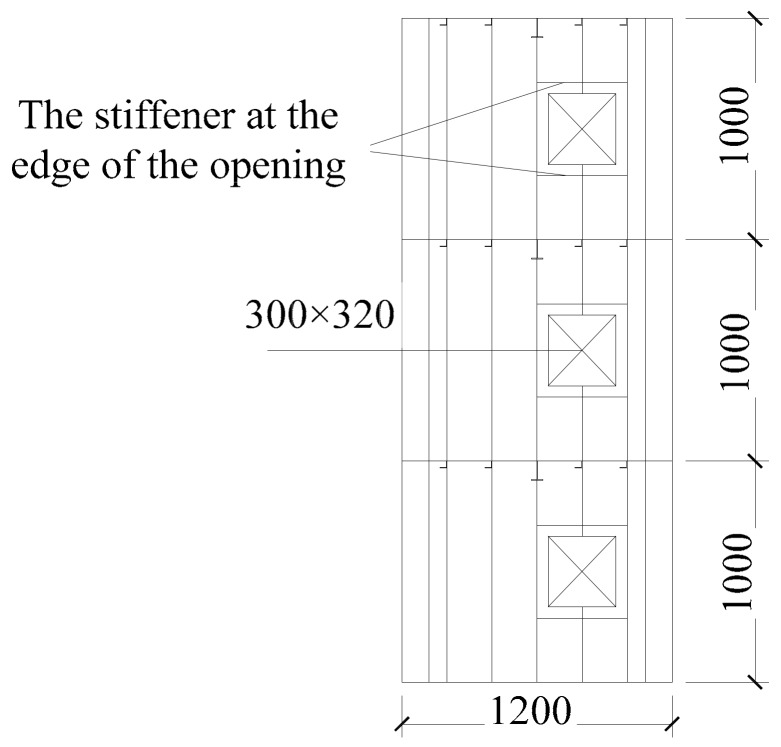
The opening position diagram.

In order to further verify the correctness of the calculation equation of the three-story single box-plate steel structure with openings space stress-bearing modular unit, the previously validated finite element analysis (FEA) model was used for finite element analysis. *χ =* 10% was selected for finite element analysis. The opening position diagram is shown in Figure 18. The value of the ultimate lateral force resistant capacity calculated by the finite element of each model was compared with that calculated by the theoretical equation, as shown in Table 4.

As could be seen from Table 4, the theoretical calculation value of the ultimate lateral force resistant capacity of the three-story single box-plate steel structure with openings space stress-bearing modular unit was in good agreement with the finite element analysis value, and the maximum deviation was less than 5.2%. Therefore, it could be considered that the calculation equation of the ultimate lateral force resistant capacity (*V_u_*_,0_) of a three-story single box-plate steel structure with openings space stress-bearing modular unit in this paper met the accuracy requirements. Therefore, Equation (9) could provide a basis for the application of box-plate steel structure with openings modular unit in practical engineering.

## 6. Conclusions and Suggestions

An experimental study was conducted to investigate the seismic performance of the box-plate steel structure with openings modular unit, and the finite element analysis (FEA) model was developed to supplement the experimental study. Finally, an equation for calculating the ultimate lateral force resistant capacity of the structure was proposed and verified by finite element simulation. The following conclusions and suggestions could be drawn within the limited range of the above research.

Both the box-plate structure with door opening and window opening modular unit had good hysteric performance and deformation ability. Within the height range of opening on each story gable wall plate, the sheared section area of the box-plate structure with window openings modular unit was smaller than that of the box-plate structure with door openings modular unit, so the ultimate lateral force bearing capacity of the box-plate structure with window openings modular unit was smaller than that of the box-plate structure door openings modular unit.

The lateral stiffness of the gable with openings could be divided into three parts in series: the lateral stiffness of steel plate wall at the upper part of the openings; the lateral stiffness of steel plate wall at the range of height of the openings; the lateral stiffness of steel plate wall at the lower part of the openings.

The failure of the box-plate structure with door openings modular unit began with buckling deformation at the four corners of the door openings, and then the T-stiffener on the gable had buckling deformation, at which time the lateral force resistant capacity of the box-plate structure with door openings modular unit reached the maximum. Then, the corner of the door opening was torn, and the corner of the box-plate structure with door opening modular unit was torn, and finally, the T-stiffener on the gable was broken.

The lateral force resistant capacity of the box-plate structure with window openings modular unit reached its maximum when cracks appeared in the corner of the window openings, and the buckling deformation appeared in the corner reinforcing structure rib. The damage of the box-plate structure with window openings modular unit began with the tearing at the corners of the window openings on upper and lower stories of the structure and the cracks at the corner reinforcing structure rib. When the box-plate structure with window openings modular unit was finally destroyed, the corner reinforcing structure rib of the structure was torn, and the four corners of the structure were completely deformed.

The strength degradation of the two specimens was relatively slow, and the stiffness degradation was continuous and uniform. The energy dissipation of the box-plate structure with window openings modular unit was higher than that of the box-plate structure with door openings modular unit. In practical engineering, corresponding measures should be taken to improve the rigidity of corner reinforcing construction measures and control the position of the opening in a reasonable position.

The equation for calculating ultimate lateral force resistant capacity was highly accurate and could be used as a reference in practical engineering. Therefore, in actual engineering, openings could be made in the gable of the box-plate steel structure according to the requirements, and then the lateral bearing capacity of the structure could be calculated according to the method provided in this paper. At the same time, measures should be taken to enhance the stiffness of the corner reinforcing structure rib, and the position of the openings should be as symmetrical as possible.

## Figures and Tables

**Table 1:** The dimensions of stiffeners on walls and plates.

Position	T-stiffener (mm)	L-stiffener (mm)
Wall	T70 × 3/35 × 4	L25 × 25 × 3
Plate	T85 × 4/50 × 5	L30 × 30 × 3

**Table 2:** Steel property test.

The Thickness of the Steel Plate	Elasticity Modulus/GPa	Yield Strength/MPa	Ultimate Strength/MPa	Fracture Strain/%	Yield Ratio
3 mm	221.959	331	487	20.1	0.68
4 mm	213.074	310	465	22.3	0.67
5 mm	199.459	304	461	24.4	0.66

**Table 3:** Characteristic loads and displacements of specimens.

No.	Loading direction	Yield load *P_y_*/kN	Yield displacement *Δ_y_*/mm	Peak load *P_m_*/kN	Limiting displacement *Δ_u_*/mm	Ductility factor *μ*
Specimen-1	Push	346.46	3.5	558.09	19.4	5.54
	pull	−307.52	−2.7	−512.5	−19.1	7.07
Specimen-2	Push	203.35	3.9	482.59	24.9	6.38
	pull	−180.28	−3.1	−443.23	−23.8	7.68

**Table 4:** Comparison of theoretical calculation value and the finite element analysis value.

No.	*M_c_*	*δ*	*t_w_*	*V_f_*	*V_p_*_,0_	*V_m_*	*V_u,_*_0_	Simulation	Deviation
	(kN·m)	(mm)	(mm)	(kN)	(kN)	(kN)	(kN)	(kN)	(%)
1	42.47	25.66	3.33	56.38	473.67	185.7	1245.8	1183.818	4.97
2	40.75	26.43	3.33	53.08	473.67	185.7	1239.2	1179.314	4.83
3	38.61	25.94	3.33	49.01	473.67	185.7	1231.06	1179.626	4.17
4	36.46	26.05	3.33	44.9	473.67	185.7	1222.84	1171.636	4.18
5	50.02	25.07	3.33	66.41	473.67	185.7	1265.86	1216.336	3.91
6	48.00	25.75	3.33	62.55	473.67	185.7	1258.14	1212.322	3.64
7	45.47	25.85	3.33	57.73	473.67	185.7	1248.5	1209.646	3.11
8	42.95	25.17	3.33	53.02	473.67	185.7	1239.08	1205.412	2.71
9	36.81	25.57	2.86	48.86	406.82	159.49	1070.85	1016.116	5.11
10	35.32	25.25	2.86	46.05	406.82	159.49	1065.23	1012.978	4.90
11	33.46	26.05	2.86	42.46	406.82	159.49	1058.05	1009.26	4.61
12	31.60	25.94	2.86	38.92	406.82	159.49	1050.93	999.186	4.92
13	43.41	29.84	2.86	57.59	406.82	159.49	1088.31	1047.026	3.79
14	41.66	29.86	2.86	54.09	406.82	159.49	1081.31	1042.698	3.57
15	39.47	29.54	2.86	49.74	406.82	159.49	1072.61	1036.782	3.34
16	37.27	29.86	2.86	45.36	406.82	159.49	1063.85	1034.032	2.80
